# Synthesis of [^18^F]Favipiravir and Biodistribution in C3H/HeN Mice as Assessed by Positron Emission Tomography

**DOI:** 10.1038/s41598-018-37866-z

**Published:** 2019-02-11

**Authors:** Thomas M. Bocan, Falguni Basuli, Robert G. Stafford, Jennifer L. Brown, Xiang Zhang, Allen J. Duplantier, Rolf E. Swenson

**Affiliations:** 1 Molecular and Translational Sciences Division, U.S. Army Medical Research Institute of Infectious Diseases, 1425 Porter St., Ft. Detrick, MD 21702 USA; 2 Cherokee Nation Assurance, 777 West Cherokee Street, Catoosa, OK 74015 USA; 3 General Dynamics Information Technology (GDIT), 3211 Jermantown Road, Fairfax, VA 22030 USA; 4 Imaging Probe Development Center, National Heart, Lung, and Blood Institute, National Institutes of Health, Bethesda, MD 20892 USA

## Abstract

Favipiravir (T705; 6-fluoro-3-hydroxypyrazine-2-carboxamide) is a pyrazine analog that has demonstrated potent antiviral activity against a broad spectrum of viruses in multiple *in vivo* disease models. To better understand the compounds anti-viral activity, assessment of the drug’s biodistribution and kinetics *in vivo* may lend insight into how best to evaluate the compound efficacy preclinically and to contribute to the design of clinical studies to take into account the compound’s pharmacokinetic distribution and kinetics. In the current study, a method for synthesis of [^18^F]favipiravir was developed and the biodistribution in mice naïve to and pre-dosed with favipiravir was assessed by PET and gamma counting of tissue samples. Fluorine-18 labeling of favipiravir was achieved in a one-pot, two-step synthesis using a commercially available precursor, methyl-5-chloroisoxazolo[4,5-b]pyrazine-3-carboxylate, with an overall radiochemical yield of 15–24%, a molar activity of 37–74 GBq/µmol in a 70 minute synthesis time. [^18^F]favipiravir tissue uptake and distribution was similar in naïve and pre-dosed mice; however, in the pre-dosed animals plasma clearance was more rapid and tissue clearance appeared to be prolonged. In conclusion, application of PET to the evaluation of favipiravir has demonstrated the importance of dosing regimen on the distribution and tissue uptake and clearance of the molecule. Favipiravir is cleared through the kidney as previously reported but the liver and intestinal excretion may also play an important role in compound elimination. Measurement of the tissue uptake of favipiravir as determined by PET may be a more important indicator of a compound’s potential efficacy than purely monitoring plasma parameters such as viremia and drug levels.

## Introduction

Favipiravir (T705; 6-fluoro-3-hydroxypyrazine-2-carboxamide) is a pyrazine analog that has demonstrated potent antiviral activity against a broad spectrum of viruses in multiple *in vivo* disease models^1^. Within cells, favipiravir is ribosylated and phosphorylated to its active form, favipiravir-ribofuranosyl-5′-triphosphate (RTP). RTP acts as a surrogate purine nucleotide as noted by *in vitro* studies that show the compound’s activity is attenuated by exogenous addition of purines. Favipiravir has *in vitro* activity against influenza viruses (A(H1N1)pdm09, A(H5N1), A(H7N9)) arenaviruses, phleboviruses, hantaviruses, flaviviruses, enteroviruses, alphaviruses, paramyxovirus, respiratory syncytial virus and noroviruses^1^. Favipiravir has also been shown to reduce mortality and decrease plasma and tissue viremia against wild-type pandemic H1N1 virus, oseltamivir resistant H275Y neuraminidase mutant virus^2^, western equine encephalitis^3^, West Nile virus^4^ and Crimean-Congo hemorrhagic fever^5^ in mice and yellow fever virus in hamsters^6^. In type-I interferon receptor knockout mice, favipiravir administered at 300 mg/kg orally at day 6 post-EBOV infection reduced viremia and biochemical parameters of disease severity and mortality, i.e., 100% survival^7^. We have also shown that favipiravir administered at 37.5–150 mg/kg orally reduced mortality 100% in mice infected with Ebola virus^8^. In addition, favipiravir given at a loading dose of 125–400 mg/kg orally followed by daily maintenance doses of 75–200 mg/kg orally while not having an effect on overall survival in Ebola infected non-human primates was shown to increase time to death and reduce plasma viremia^9^. In contrast, 83% of the non-human primates administered favipiravir at 250 mg/kg intravenously (iv) on day 0 followed by 150 mg/kg iv on days 1–13 and infected with Marburg virus survived^9^. Based on the published reports, one can conclude that favipiravir, albeit at high doses, reduces viral replication and mortality in rodent models of viral infection.

The objective of the present investigation was to develop a route for synthesis of [^18^F]favipiravir and to characterize the biodistribution dynamically using PET. At defined time points post-intravenous injection of [^18^F]favipiravir, organ samples were subjected to gamma counting to verify tissue distribution. Since clinical dosing of favipiravir required a loading dose followed by a maintenance dose, we evaluated the distribution of [^18^F]favipiravir in mice naïve to favipiravir and after 7 doses of favipiravir. The JIKI trial which treated Ebola-infected patients with favipiravir utilized a loading dose of 6000 mg on day 1 followed by a maintenance dose of 1200 mg twice daily for the remainder of the study^10^. The authors selected the dosing regimen based on modeling of pharmacokinetic data which predicted stable plasma drug levels; however, the clinical data indicated that after 4 days of dosing plasma drug levels were lower than calculated by the model. Given that the study was only able to demonstrate changes in plasma levels following treatment, we hypothesized that a loading dose of favipiravir would result in different distribution patterns of the drug and that PET would allow us to detect the differences dynamically.

## Results

**Figure 1:**
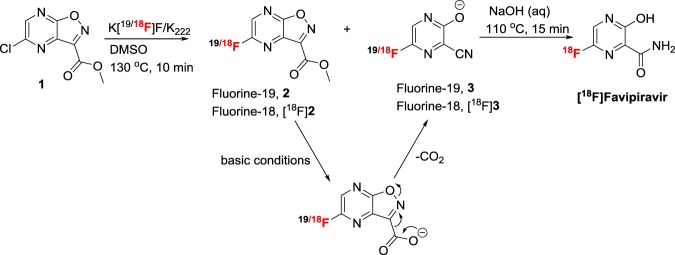
Synthesis of fluorine-18 labeled favipiravir.

**Figure 2:**
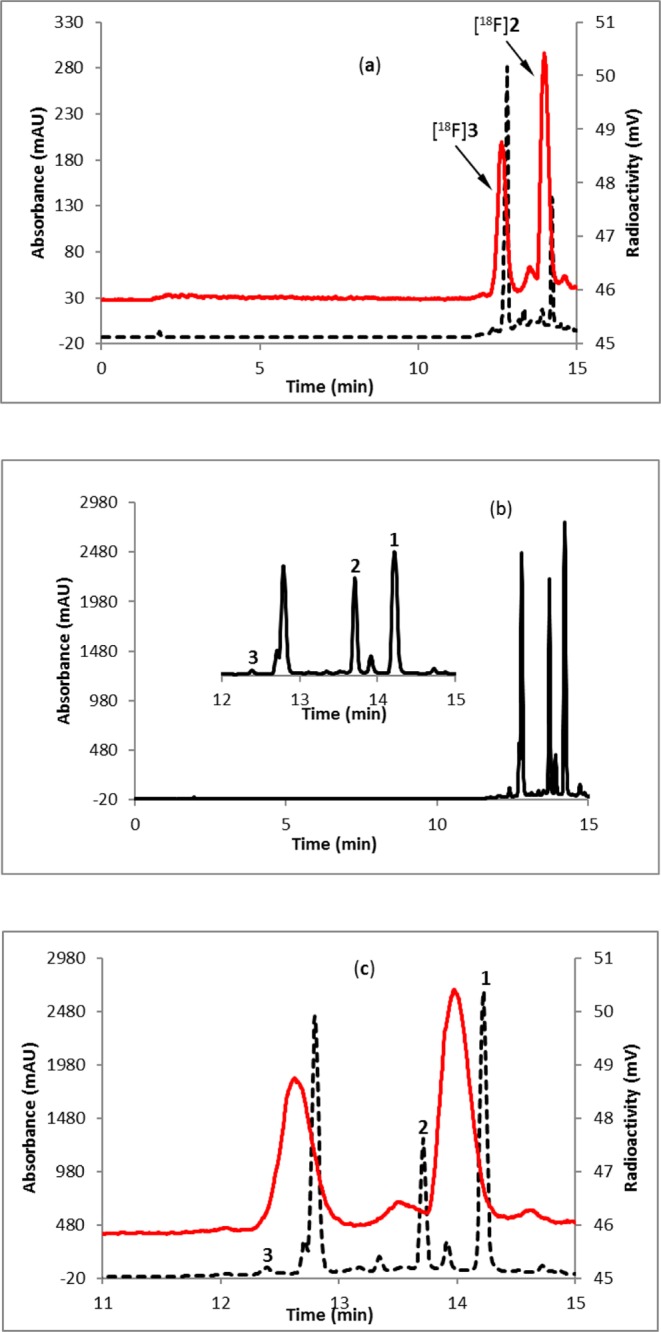
HPLC profile of reaction mixture of (**a**) [^18^F]**2** and [^18^F]**3** (fluorine-18 reaction); (**b**) **2** and **3** (fluorine-19 reaction); (**c**) coinjection of fluorine-18 reaction mixture with fluorine-19 reaction mixture. Solid line, in-line radiodetector; dashed line, UV detector at 254 nm.

**Figure 3:**
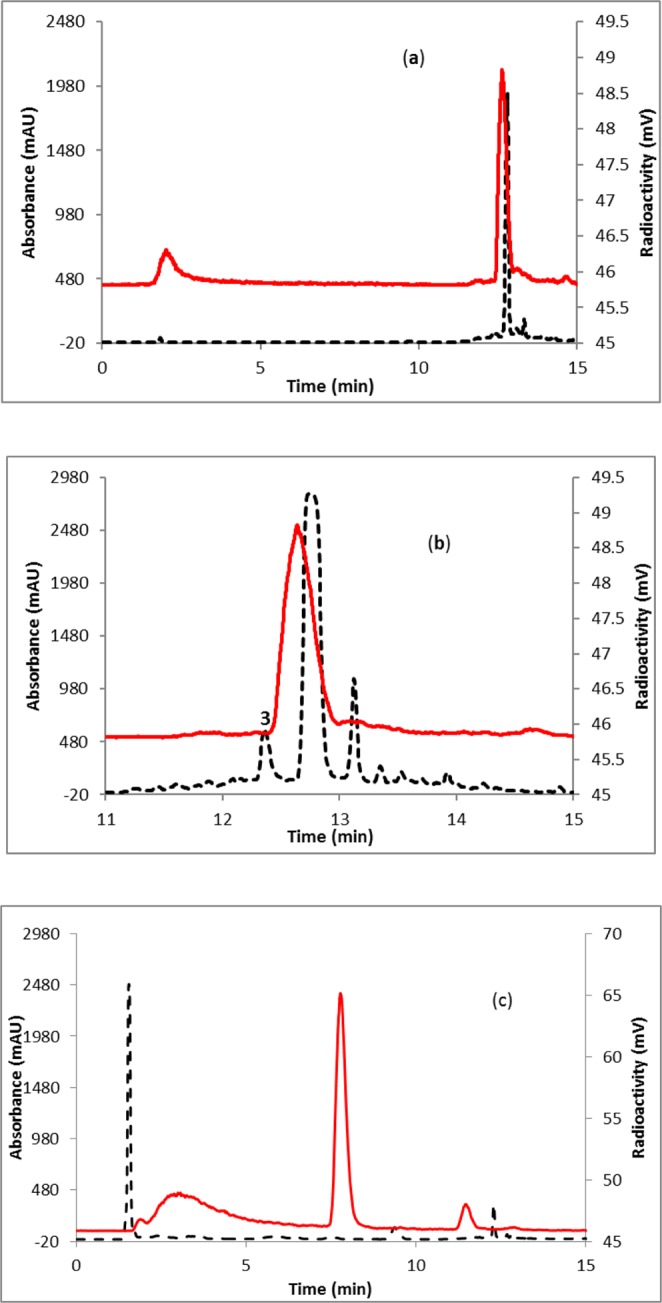
HPLC profile of reaction mixture of (**a**) [^18^F]**3**; (b) [^18^F]**3** coinjected with **3**; (**c**) [^18^F]favipiravir. Solid line, in-line radiodetector; dashed line, UV detector at 254 nm.

**Figure 4:**
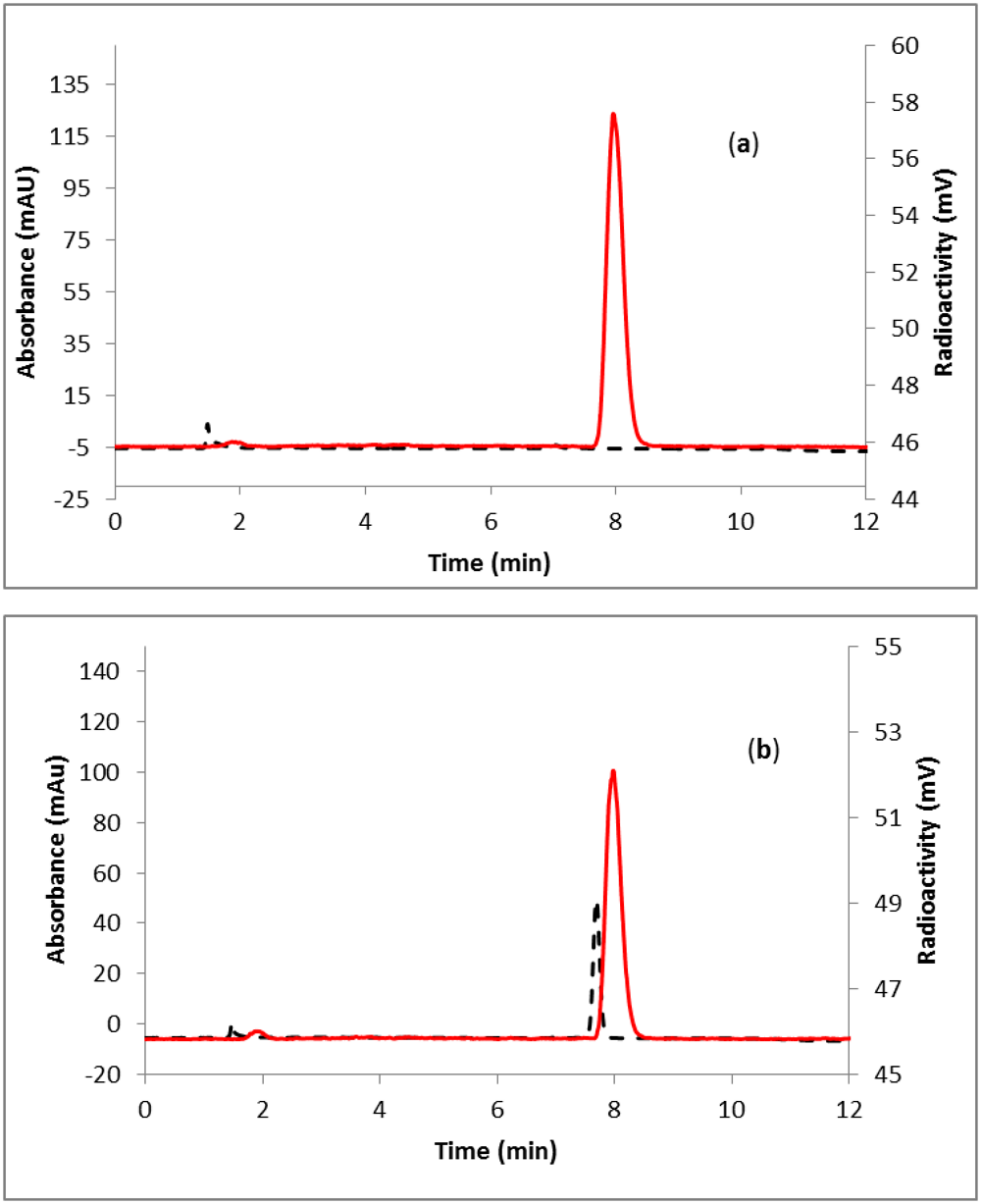
HPLC profile of (**a**) purified [^18^F]favipiravir; (**b**) purified [^18^F]favipiravir co-injected with the non-radioactive standard favipiravir. Solid line, in-line radiodetector; dashed line, UV detector at 254 nm.

Fluorine-18 labeling of favipiravir was achieved in a one-pot, two-step (Fig. 1) synthesis using a commercially available precursor, methyl-5-chloroisoxazolo[4,5-b]pyrazine-3-carboxylate (**1**, Fig. 1). Labeling efficiency was first standardized manually using starting activity of 0.37–3.7 GBq. The progress of the reaction was monitored by radio-TLC (see Supplementary Fig. S1), supporting a 76% consumption of fluorine-18 at 130 °C for 10 minutes. Analysis of crude reaction mixture by HPLC revealed the formation of two major labeled intermediates, [^18^F]**2** and [^18^F]**3** (Fig. 2a). To identify the intermediates, fluorination reaction of the precursor (**1**) was performed with fluorine-19 and analyzed by HPLC (Fig. 2b). The individual peaks were characterized by mass spectrometry, supporting the formation of 5-fluoroisoxazolo[4,5-b]pyrazine-3-carboxylic acid (**2**) and 3-cyano-5-fluoropyrazin-2-olate (**3**). The latter anionic compound **3** is stabilized by the electron-withdrawing nature of the adjacent cyano group, as well as a keto-enol interconversion between 2- hydroxypyrazine and pyrazin-2-one. The other major byproduct of this reaction was 5-fluoroisoxazolo[4,5-b]pyrazine-3-carboxylic acid. This nonradioactive fluorination reaction mixture was co-injected in an HPLC with the fluorine-18 labeled reaction mixture to confirm the formation of labeled intermediates, [^18^F]**2** and [^18^F]**3** (Fig. 2c). The intermediate [^18^F]**2** was immediately converted to intermediate [^18^F]**3** upon addition of 1N sodium hydroxide at room temperature (Fig. 3a) and the identity was confirmed by coinjection with nonradioactive (fluorine-19) reaction mixture (Fig. 3b). The quantitative conversion of the intermediates ([^18^]**2** and [^18^F]**3**) to the final product, [^18^F]favipiravir, was observed when the reaction mixture was heated at 110 °C for 15 minutes with 1N sodium hydroxide (Fig. 2c). The compound was purified by HPLC to produce >98% radio-chemically pure [^18^F]favipiravir (Fig. 4a). The overall radiochemical yield was 15–24% (uncorrected, n = 9) in a 70 minute synthesis time. The identity of the product, [^18^F]favipiravir, was confirmed by comparing its HPLC retention time with co-injected authentic nonradioactive favipiravir (Fig. 4b).

After successful manual standardization of the procedure a fully automated synthesis was performed in a GE Tracerlab FX-N module (see Supplementary Fig. S2). The overall radiochemical yield was comparable with the manual synthesis with a molar activity of 37–74 GBq/µmol (n > 5).

**Figure 5:**
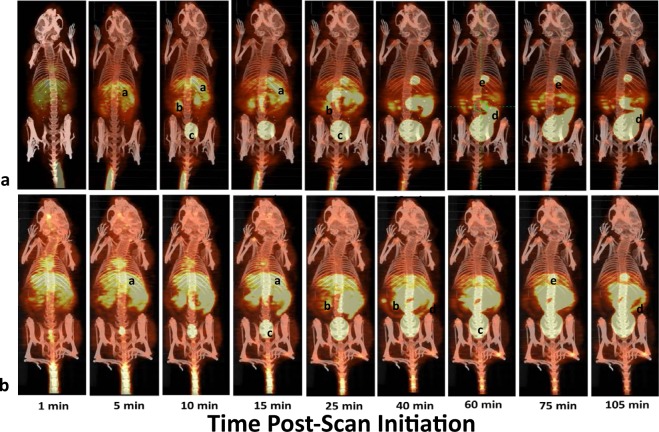
Dynamic [^18^F]favipiravir kinetics in naïve (**a**) and pre-dosed (**b**) mice as assessed by PET. Labels: (**a**) Liver; (**b**) Kidney; (**c**) Urinary Bladder; (**d**) Intestine; (**e**) Gall Bladder.

The dynamic PET scans of naïve animals showed rapid uptake in the liver and gall bladder followed by the appearance of tracer in the kidney and urinary bladder (Fig. 5a). Over time, radiotracer levels were noted at various segments of the intestinal tract. In animals pre-dosed with favipiravir, radiotracer distribution was similar to that noted for the naïve animals (Fig. 5b); however, a more rapid uptake in the liver and a more intense signal in the brain and muscle were noted.

**Figure 6:**
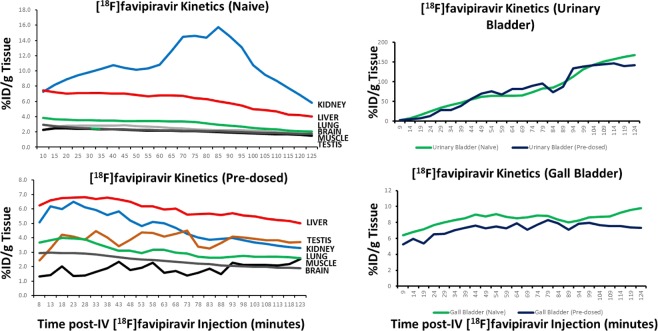
Quantification of PET kinetics of [^18^F]favipiravir uptake in kidney, liver, lung, brain, muscle and testis over 2 hours in naïve and mice pre-dosed with favipiravir. Kinetics of [^18^F]favipiravir uptake in the urinary bladder and gall bladder over time in naïve and mice pre-dosed with favipiravir as assessed by PET.

Upon quantification of the PET scanning results, [^18^F]favipiravir in mice naïve to favipiravir showed a rapid uptake of tracer in the kidney, i.e., 8–16% ID/g, which remained above 6% ID/g throughout the time course of imaging (Fig. 6). In contrast in mice pre-dosed with favipiravir, kidney [^18^F]favipiravir levels were less than those noted for naïve animals and remained relatively constant and between 4–6% ID/g over the course of the study. Liver [^18^F]favipiravir levels for both treatment groups were approximately 6% ID/g and remained relatively constant over the 2 h imaging session (Fig. 6). Lung, brain, abdominal muscle and testis levels of [^18^F]favipiravir were comparable, i.e., 2–4% ID/g, among the naïve and pre-dosed groups over the course of the imaging session. Urinary bladder and gall bladder radioactivity levels in both naïve and pre-dosed animals increased from 0 to approximately 150% ID/g and 5 to 9% ID/g, respectively (Fig. 6).

**Figure 7:**
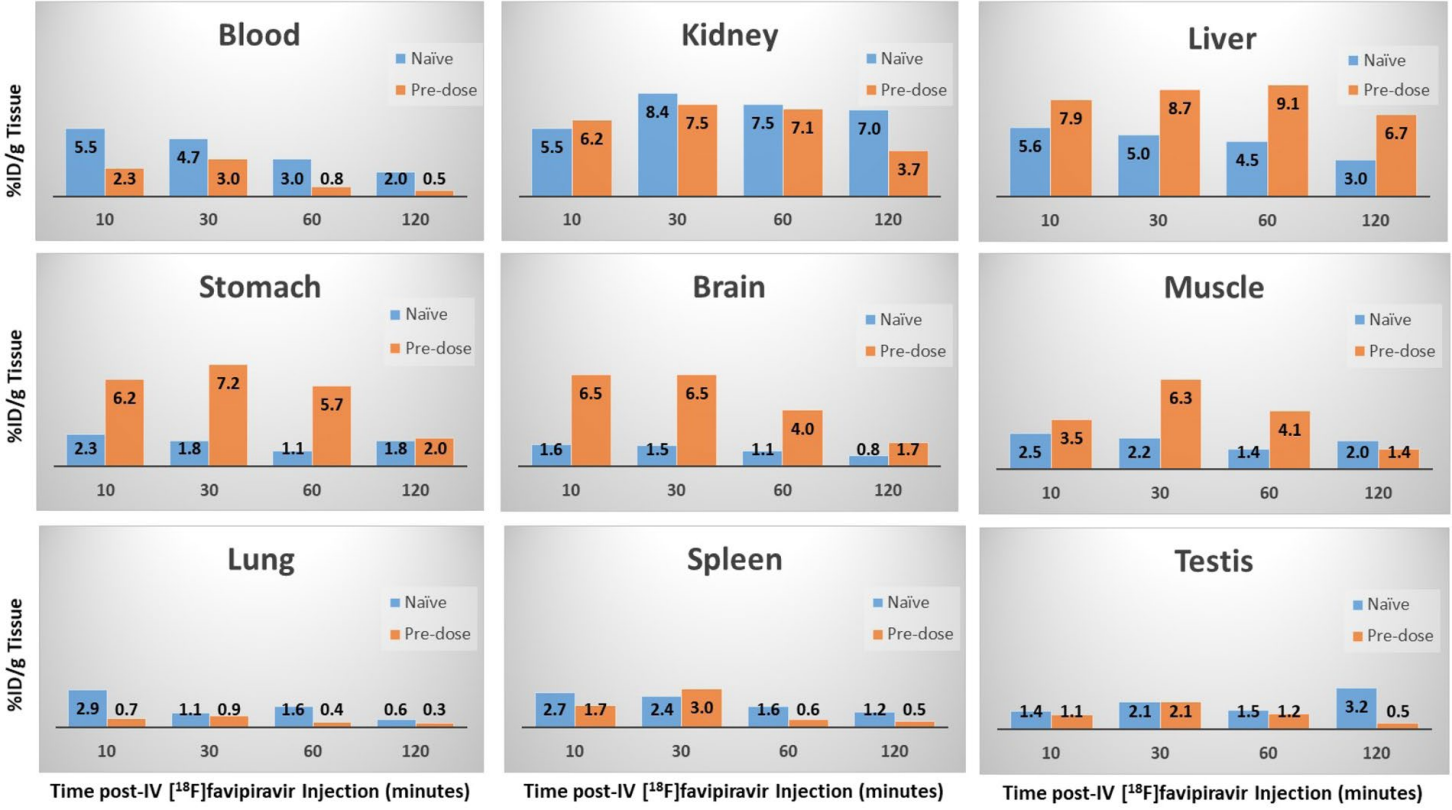
Quantification of the tissue distribution and temporal changes of [^18^F]favipiravir by gamma counting in naïve and pre-dosed mice. Value in each bar represents the % ID/gm for the respective tissue.

The results from the gamma counter studies verified the PET observations; however, some additional insights and differences between the naïve and pre-dosed animals were noted. Blood radioactivity cleared rapidly in the pre-dosed animals and was approximately 25–50% of the amount noted in the blood of naïve animals over the 4 time points evaluated (Fig. 7). Kidney levels of radiotracer were similar among the two groups with a slight increase at 30 and 60 minutes post-dose as noted in the dynamic PET scan. Liver, stomach, brain and muscle levels of radioactivity were approximately 2- to 5-fold higher in the pre-dosed animals relative to the naïve animals (Fig. 7). Comparable amounts of radioactivity were noted in the lung, spleen, testis, heart and seminal vesicles, i.e., 1–3% ID/g. Upon examination of the intestine, it was apparent that the bulk of radioactivity was in the luminal contents (see Supplementary Fig. S3) which is consistent with the observations from the dynamic PET scan showing movement of the radiotracer apparently with peristalsis. Markedly higher levels of radioactivity were present in the luminal contents of the large intestine of the naïve animals, i.e., 5–13% ID/g, relative to the pre-dosed animals while the intestinal wall levels of radioactivity were similar among the groups.

## Discussion

A simple, one-pot, two-step automated radiolabeling method was developed to produce [^18^F]favipiravir with an overall radiochemical yield of 15–24% in a 70 minute synthesis time. [^18^F]Favipiravir tissue uptake and distribution was similar in naïve and pre-dosed mice; however, in the pre-dosed animals plasma clearance was more rapid and tissue clearance appeared to be prolonged. Dynamic PET scanning which was verified through measurement of tissue radioactivity by gamma counting proved to be a sensitive tool for tracking [^18^F]favipiravir distribution.

The radiolabeling of favipiravir with fluorine-18 for the first time provides a tool for better understanding the tissue distribution, kinetics of uptake and clearance and differential effects related to treatment, e.g., single dose versus pre-loading. Initially, we evaluated the direct fluorine-19 displacement of a protected form of 2-hydroxy-5-chloro-3-carboxamide or other suitable leaving group, and were unable to obtain the desired fluorinated product. In the patent application, Nakamura *et al*.^11^ reported the synthesis of non-radioactive favipiravir using a commercially available starting material, methyl 5-chloroisoxazolo[4,5-b]pyrazine-3-carboxylate (**1**). Their method has been adopted with modifications for the preparation of fluorine-18 labeled favipiravir. The synthetic route utilizes the same starting material, which allows the direct nucleophilic aromatic substitution of chloride with [^18^F]fluoride. During the fluorination reaction, the methyl ester was lost due to decarboxylation with homolysis of the nitrogen oxygen bond to form the nitrile intermediate ([^18^F]**3**, Fig. 1). The compound [^18^F]**3** was then hydrolyzed with sodium hydroxide to the desired labeled favipiravir. The synthetic route employed allowed for rapid synthesis with high yield, purity and molar activity and allowed us to automate the procedure for routine synthesis.

Administration of [^18^F]favipiravir to naïve mice results in rapid uptake and clearance through liver, kidney and intestine. Tissue retention of favipiravir is dependent on the compound being ribosylated and phosphorylated. The imaging data suggests that the rate of tissue clearance may be faster than the rate of ribosylation and/or phosphorylation leading to clearance through the liver via the gall bladder and intestine either as the parent or hydroxylated metabolite or through the kidney potentially due to metabolism. Favipiravir has been reported to be metabolized by aldehyde oxidase and the hydroxylated metabolite/s are excreted by way of the kidney^12^. The PET data following a single injection of [^18^F]favipiravir is consistent with that observation; however, it appears as though the liver may also play a role in the rapid excretion of favipiravir. Rapid uptake in the liver was noted and within 15–20 minutes post-injection radiotracer was noted in the gall bladder and segments of the intestinal tract. It is unknown whether the observed radioactivity in gall bladder and intestine is the parent or a metabolite since the fluorine-18 would be present on both molecules.

Following exposure to a loading pre-dose of 250 mg/kg bid on day 1 and twice daily doses of 150 mg/kg, [^18^F]favipiravir clearance from the blood and distribution across the body was more rapid than that noted in animals naïve to favipiravir. Marked liver uptake relative to other organs was noted within 5 minutes of radiotracer administration and a broader whole body distribution of drug is observed, e.g., brain and muscle. The difference in clearance and tissue uptake relative to that observed after a single dose may be related to metabolism and activation of the drug. Favipiravir inhibits aldehyde oxidase *in vitro* and the pharmacokinetic profile was consistent with auto-inhibition of the metabolizing enzyme following chronic dosing^12^. Chronic dosing of favipiravir results in plasma drug levels consisting of 90% parent and 10% of the hydroxylated metabolite (M1) (Personal communication, Dr. Robert Lenk, FUJIFILM Pharmaceuticals USA) while after a single dose there are equal concentrations of parent and metabolite M1. Since the cellular uptake of favipiravir is dose-dependently related to the extracellular concentration^13^, the extracellular to intracellular favipiravir concentration gradient may be higher under the pre-dosing conditions promoting greater intracellular pools of the ribosylated and phosphorylated form. Thus, under the pre-dosing experimental design higher plasma drug levels of the parent may promote greater cellular uptake resulting in phosphorylation to favipiravir-ribofuranosyl-5′-triphosphate (favipiravir-RTP) and trapping of the active form of favipiravir in the cell and such an event may be reflected in the PET studies as a broader tissue distribution and more prolonged uptake of [^18^F]favipiravir.

With chronic dosing of favipiravir, tissue levels of favipiravir-RTP were observed indicating a conversion of favipiravir to the active anti-viral moiety^8^. After 5 days of orally administered 300 mg/kg favipiravir, favipiravir-RTP levels were 0.5, 0.1 and 0.05 pmoles/g tissue in the muscle, liver and kidney, respectively^8^. After 24 h following cessation of dosing, favipiravir-RTP levels were undetectable which suggests relatively rapid dephosphorylation and clearance. The PET results showing a more broad distribution of [^18^F]favipiravir under pre-dosed conditions is consistent with the observation of tissue favipiravir-RTP levels. One might speculate that chronic dosing reduces metabolism and clearance of favipiravir resulting in an increased plasma pool of compound available for tissue uptake, ribosylation and phosphorylation which is depicted as a broader distribution and reduced clearance in the PET experiment. When plasma drug levels become reduced as one would expect following cessation of dosing, the flux of compound from the tissue to blood results in more rapid clearance as noted in the PET experiment under naïve conditions.

The results of the current investigation taken together with published work demonstrates that PET imaging may provide information on the dynamic distribution of favipiravir that can aid in the design of clinical studies and better interpret the pharmacokinetic/pharmacodynamic effects of a compound. It is tempting to speculate how the results of the current investigation would have impacted the preclinical and clinical evaluation of favipiravir if the data were known prior to compound advancement. Formulations or chemical modifications to enhance uptake and reduce clearance or metabolism could have potentially reduced the dose required and eliminated the rapid clearance noted by the PET studies. Alternatively, various pre-dosing regimens, e.g., preloading dose required to achieve parent plasma drug levels, or formulations, e.g., intravenous predose followed by oral administration, may have improved overall exposure and promoted formation of intracellular RTP levels, the active moiety of favipiravir, and thereby alter the clearance and distribution noted in the PET studies. Thus, if the PET results were known sooner, comparison of dosing paradigms and formulations designed to alter drug metabolism using the radiolabeled favipiravir may have provided data for more efficient assessment of drug efficacy.

In conclusion, the application of PET to the evaluation of favipiravir has demonstrated the importance of dosing regimen on the distribution and tissue uptake and clearance of the molecule, i.e., naïve versus pre-dosing paradigm. Favipiravir is cleared through the kidney as previously reported^12^ but the liver and intestinal excretion may also play an important role in compound elimination as noted by the dynamic PET scans. Finally, measurement of the tissue uptake of favipiravir as determined by PET may be a more important indicator of a compound’s potential efficacy than purely monitoring plasma parameters such as viremia and drug levels.

## Materials and Methods

### Synthesis of [^18^F]favipiravir

#### Materials

The precursor for radiolabeling of favipiravir, methyl-5-chloroisoxazolo[4,5-b]pyrazine-3-carboxylate (**1**), was obtained from Combi-Blocks (San Diego, CA, USA). The fluoro intermediate, 5-fluoroisoxazolol4,5-bipyrazine-3–3carboxylic acid (**2**) was purchased from ASTATECH (Bristol, PA, USA). All other chemicals and solvents were received from Sigma-Aldrich (St. Louis, MO, USA) and used without further purification. Fluorine-18 was obtained from the National Institutes of Health cyclotron facility (Bethesda, MD, USA). Chromafix 30-PS-HCO_3_ anion-exchange cartridges were purchased from Macherey-Nagel (Düren, Germany). High-resolution mass spectra (HRMS) were collected on a Waters Xevo G2-XS QTof (ESI mode) mass spectrometer. The semi-preparative and analytical columns were obtained from Phenomenex (Torrance, CA, USA). iTLC-SG plates were obtained from Agilent Technologies (Santa Clara, CA, USA). Semi-prep HPLC purification and analytical HPLC analyses for radiochemical work were performed on an Agilent 1200 Series instrument equipped with multi-wavelength detectors along with a flow count radiodetector (Eckert & Ziegler, B-FC-3500 diode). iTLC-SG papers were developed using acetonitrile. The papers were read in an Eckert & Ziegler TLC scanner (B-AR2000-1). The fully automated synthesis was performed in a GE Tracerlab FX-N Pro module. HPLC conditions: purification: Phenomenex Luna (2) C18 column, 5 μm, 10 × 250 mm; 5% ethanol in 50 mM H_3_PO_3_, 4 mL/minute. Analytical HPLC conditions:

Phenomenex Lun (2) C18 column, 5 μm, 4.6 × 100 mm; 1–5% B in 0–8 minutes, 5–95% B in 8–15 minutes; A = acetonitrile (0.1% TFA), B = water (0.1% TFA), 1 mL/minute.

#### Manual [^18^F]favipiravir synthesis method

Methyl-5-chloroisoxazolo[4,5-b]pyrazine-3-carboxylate (10 mg) in dimethyl sulfoxide (DMSO, 0.3 mL) was added to the azeotropically dried mixture of [^18^F]KF/K_222_ (2 mg K_2_CO_3_, 10 mg K_222_) and heated at 130 °C for 10 minutes. The reaction mixture was cooled for 2 minutes and reacted with 1N sodium hydroxide (400 μL) followed by heating at 110 °C for 15 minutes. The solution was diluted with 0.2 M H_3_PO_4_ (3 mL) and purified by HPLC using a semi-prep column.

#### Nonradioactive fluorination

To the mixture of methyl-5-chloroisoxazolo[4,5-b]pyrazine-3-carboxylate (**1**, 20 mg, 0.09 mmol), KF (25 mg, 0.43 mmol), K_222_ (10 mg, 0.03 mmol) was added to 1 mL DMSO and heated at 130 °C for 10 minutes. The reaction mixture was analyzed by HPLC (Fig. 2b) and LC-MS. The compounds were not isolated. HRMS (ESI) calculated mass for the parent precursor (**1**) C_7_H_5_ClN_3_O_3_ 214.0013 [M + H]^+^, found 214.0013 [M + H]^+^; compound (**2)** C_7_H_5_FN_3_O_3_ 198.0312 [M + H]^+^, found 198.0313 [M + H]^+^; compound (**3)** C_5_H_3_FN_3_O 140.0255 [M + H]^+^, found 140.0260 [M + H]^+^; byproduct 5-fluoroisoxazolo[4,5-b]pyrazine-3-carboxylic acid C_5_H_3_ClN_3_O 1565.9959 [M + H]^+^, found 155.9969 [M + H]^+^.

#### Synthesis of compound **3**

To the solution of compound **2** (200 mg, 1 mmol) in acetonitrile (1 mL) was added 1N NaOH solution (1 mL, 1 mmol) and the mixture was stirred at room temperature for 3 h. To the solution was added 1N HCl (1 mL) and water (200 mL). Product was extracted with EtOAc (3 × 100 mL), and dried over anhydrous MgSO_4_ and filtered. The filtrate was concentrated, and solid residue was washed with hexane (10 mL) to obtain compound **3** as yellow solid (122 mg, 0.87 mmol, 87%). HRMS (ESI) calculated mass for the parent C_5_H_3_FN_3_O 140.0255 [M + H]^+^, found 140.0260 [M + H]^+^. ^1^H NMR (400 MHz, DMSO-*d*_6_) δ 13.67 (s, 1 H), 8.53 (d, *J* = 7.6 Hz, ^1^H). ^13^C NMR (101 MHz, DMSO-*d*_6_) δ 160.66, 152.79 (d, *J* = 241.7 Hz), 135.18 (d, *J* = 41.4 Hz), 114.63, 111.33. ^19^F NMR (376 MHz, DMSO-*d*_6_) δ -93.77^11^.

#### Automated [^18^F]favipiravir synthesis method

Azeotropic drying of [^18^F]fluoride was performed by passing 11.1 GBq [^18^F]fluoride in 2.5 mL of target water through a PS-HCO_3_ cartridge followed by rinsing with 1 mL acetonitrile. [^18^F]fluoride was eluted from the cartridge into Reactor 1 with the eluent (2 mg K_2_CO_3_, 10 mg K_222_ in 1 mL methanol and 200 µL water) from vial 1. Methanol was selected as an eluent over acetonitrile for better elution efficiency of [^18^F]fluoride from PS-HCO_3_ cartridge^14^. The mixture was concentrated under N_2_/vacuum at 75 °C for 4 minutes. Reactor 1 was cooled to 50 °C, acetonitrile from vial 2 was added and the active material was azeotropically dried at 55 °C for 3 minutes, then at 95 °C for 3 minutes under N_2_/vacuum. The radioactive material was further dried using a vacuum for 3 minutes. The total [^18^F]fluoride drying cycle took about 20 minutes. To the azeotropically dried mixture of [^18^F]KF/K_222_ in Reactor 1 was added methyl-5-chloroisoxazolo[4,5-b]pyrazine-3-carboxylate (10 mg) in DMSO (0.3 mL) from vial 3. The mixture was heated at 130 °C for 10 minutes. The reaction mixture was cooled to 55 °C and 400 µL of 1N sodium hydroxide from vial 4 was added, followed by heating at 110 °C for 15 minutes. The solution was cooled to 50 °C and neutralized with 0.2 M H_3_PO_4_ solution (3 mL) from vial 5. The neutralized mixture in Reactor 1 was transferred to tube 2 and injected into the HPLC for purification. The collected product was buffered to pH ~7 with 45 mM sodium phosphate (see Supplementary Fig. 2).

### Experimental Design

Two separate experiments were performed to dynamically assess the biodistribution of [^18^F]favipiravir. In the first study termed “naïve”, 3 male C3H/HeN mice were administered 250 µCi (9.25 MBq) [^18^F]favipiravir in sodium phosphate buffer, pH 7.0, by tail vein injection, anesthetized and immediately placed on the Siemens Inveon preclinical PET/CT system (Siemens Medical Solutions, Knoxville, TN). In the second study termed “pre-dosed”, 3 male C3H/HeN mice were dosed with 250 mg/kg favipiravir in 0.4% methylcellulose, po, bid on day 1, 150 mg/kg favipiravir in 0. 4% methylcellulose, po, bid on days 2 and 3 and 150 mg/kg favipiravir in 0.4% methylcellulose, po, qd on day 4 at 0800 h. On day 4, the mice were administered 250 µCi (9.25 MBq) [^18^F]favipiravir by tail vein injection at 1300 h, anesthetized and immediately placed on the Siemens Inveon preclinical PET/CT system. The average time between receiving the tail vein injection and initiation of PET scan was 4 minutes. The mice underwent a dynamic 2 h PET scan followed by a 5 minute CT scan. Upon completion of the PET/CT scan, the mice from both studies were heavily sedated under isoflurane anesthesia, the chest cage was opened, a sample of blood was taken by cardiac puncture and the animals were perfused with 30 ml of phosphate buffered saline (PBS) until the outflow from the right atrium ran clear. The blood sample and the excised organs were placed in tubes and then into a Perkin-Elmer 2480 automated gamma counter (Singapore) for assessment of organ radioactivity at 2 h. Blood, kidney, liver, stomach, brain, abdominal muscle, lung, spleen, testis, heart, seminal vesicle, small intestine, large intestine and intestinal contents were removed for radiotracer quantification by gamma counting. In separate cohorts of 2 animals each from the “naïve” and “pre-dosed” studies, mice were administered 250 µCi (9.25 MBq) [^18^F]favipiravir by tail vein injection and necropsied 10, 30, and 60 minutes post-radiotracer administration and the organs were placed in the Perkin-Elmer gamma counter. Like for the 2 h samples from the animals undergoing PET imaging, the organs were cleared with PBS by whole body perfusion prior to removal. The gamma counter data were reported in terms of percent injected dose per gram of tissue (% ID/g), calculated as a ratio of tissue radioactivity concentration (Bq/g) to total injected activity (Bq) corrected to time of iv injection.

Research was conducted under an IACUC approved protocol in compliance with the Animal Welfare Act, PHS Policy, and other Federal statutes and regulations relating to animals and experiments involving animals. The facility where the research was conducted is accredited by the Association for Assessment and Accreditation of Laboratory Animal Care, International and adheres to principles stated in the Guide for the Care and Use of Laboratory Animals, National Research Council, 2011.

### PET/CT Imaging and Data Acquisition

PET/CT scanning was performed using an Inveon preclinical PET/CT system (Siemens Medical Solutions, Knoxville, TN) with a spatial resolution of ~1.5 mm full width at half maximum at the center of the field of view. Mice were placed in a restrainer and administered 250 µCi (9.25 MBq) of [^18^F]favipiravir in ~150 µL volume via IV tail vein injection and immediately anesthetized with isoflurane (4% induction, 2% maintenance) delivered in oxygen. The mice were positioned in the center of the PET field of view and PET imaging initiated for a period of 2 h. Upon completion of the PET imaging session, a 5 minute CT scan (80 kV, 500 µA, 98 µm, and 360° rotation in 220 steps) was initiated. During all imaging procedures, animal respiration rate and body temperature were monitored and maintained using an M2M-BioVet™ small animal physiological monitoring system (M2M imaging, Cleveland, OH). When scanning was completed, mice were heavily sedated under isoflurane and organs removed as described above for assessment of radiotracer distribution by gamma counting.

### Image Reconstruction and Data Analysis

All image reconstructions were performed using Siemens’ Inveon Acquisition Workplace v2.1 software package (Siemens Medical Solutions, Knoxville, TN). Hounsfield Unit (HU) calibrated CT data were reconstructed using a Feldkamp reconstruction algorithm with a Shepp-Logan reconstruction filter, slight noise reduction, and beam hardening correction applied. Decay-corrected PET images were reconstructed using an iterative OSEM3D/MAP algorithm with scatter, dead time, and CT-based attenuation correction. The reconstruction parameters were 18 subsets, 3 iterations in a 128 × 128 matrix with a target resolution of 1.2 mm. PET images were coregistered to corresponding CT data using VivoQuant v2.5 image processing software (inviCRO, LLC, Boston, MA). The PET imaging data were summed in bins of 5 minute intervals over the entire 120 minute scan and graphically represented. Volumes of interest were defined within the kidney, liver, urinary bladder, gall bladder, lung, testis, brain and abdominal muscle and the amount of radioactivity was quantified at intervals over the 120 minute scan. PET imaging data were reported in terms of percent injected dose per gram of tissue (% ID/g), calculated as a ratio of tissue radioactivity concentration (Bq/g) at time of scan to total injected activity (Bq) at time of scan.

### Disclaimer

Opinions, interpretations and conclusions are those of the authors and not necessarily endorsed by the U.S. Army.

## Supplementary information

Supplemtary Information

## Data Availability

All data generated or analyzed during this study are included in this published article (and its Supplementary Information files).
