# Supplementary material for: Synthesis of [18F]Favipiravir and Biodistribution in C3H/HeN Mice as Assessed by Positron Emission Tomography
Source: Sci Rep. 2019 Feb 11;9:1785. doi: 10.1038/s41598-018-37866-z (PMC6370782; doi:10.1038/s41598-018-37866-z)
Supplement: Supplementary file 1 — Supplemtary Information [file 41598_2018_37866_MOESM1_ESM.pdf]

Synthesis of [^{18}F]Favipiravir and Biodistribution in C3H/HeN Mice as Assessed by Positron Emission Tomography

Thomas M. Bocan[1,2*], Falguni Basuli[4], Robert G. Stafford[1], Jennifer L. Brown[1,3], Xiang Zhang[4], Allen J. Duplantier[1,2], and Rolf E. Swenson[4]

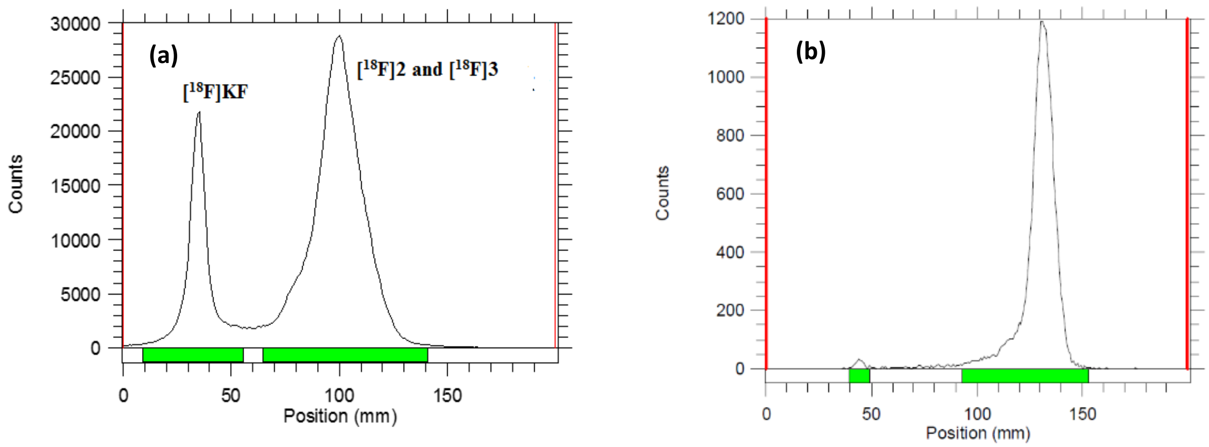

(a)

Reg	(mm) Start	(mm) Stop	(mm) Centroid	RF	Region Counts	Region CPM	% of Total	% of ROI
Rgn 1	9.2	56.0	35.2	0.176	273930.0	273930.0	23.83	24.38
Rgn 2	64.6	141.7	99.2	0.496	849556.0	849556.0	73.92	75.62
2 Peaks					1123486.0	1123486.0	97.76	100.00

(b)

Reg	(mm) Start	(mm) Stop	(mm) Centroid	RF	Region Counts	Region CPM	% of Total	% of ROI
Rgn 1	39.5	49.0	44.3	0.221	177.0	177.0	0.95	0.96
Rgn 2	93.2	152.9	129.8	0.649	18173.0	18173.0	97.17	99.04
2 Peaks					18350.0	18350.0	98.12	100.00

Supplementary Figure 1: Radio-TLC chromatogram of (a) the radiofluorination reaction; and (b) the purified [^{18}F]favipiravir.

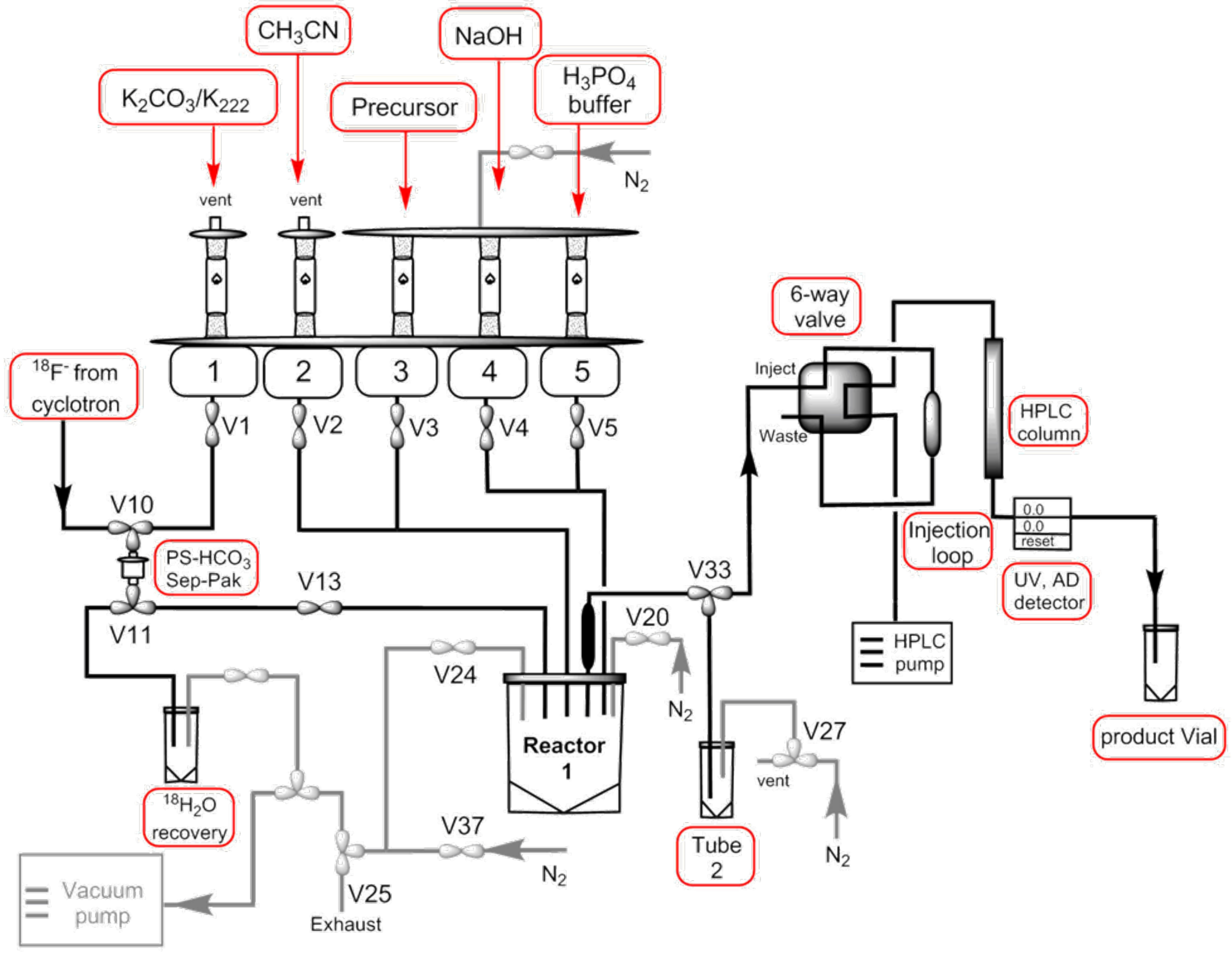

Supplementary Figure 2: Schematic diagram of the automated synthesis on GE FX-N Pro Module.

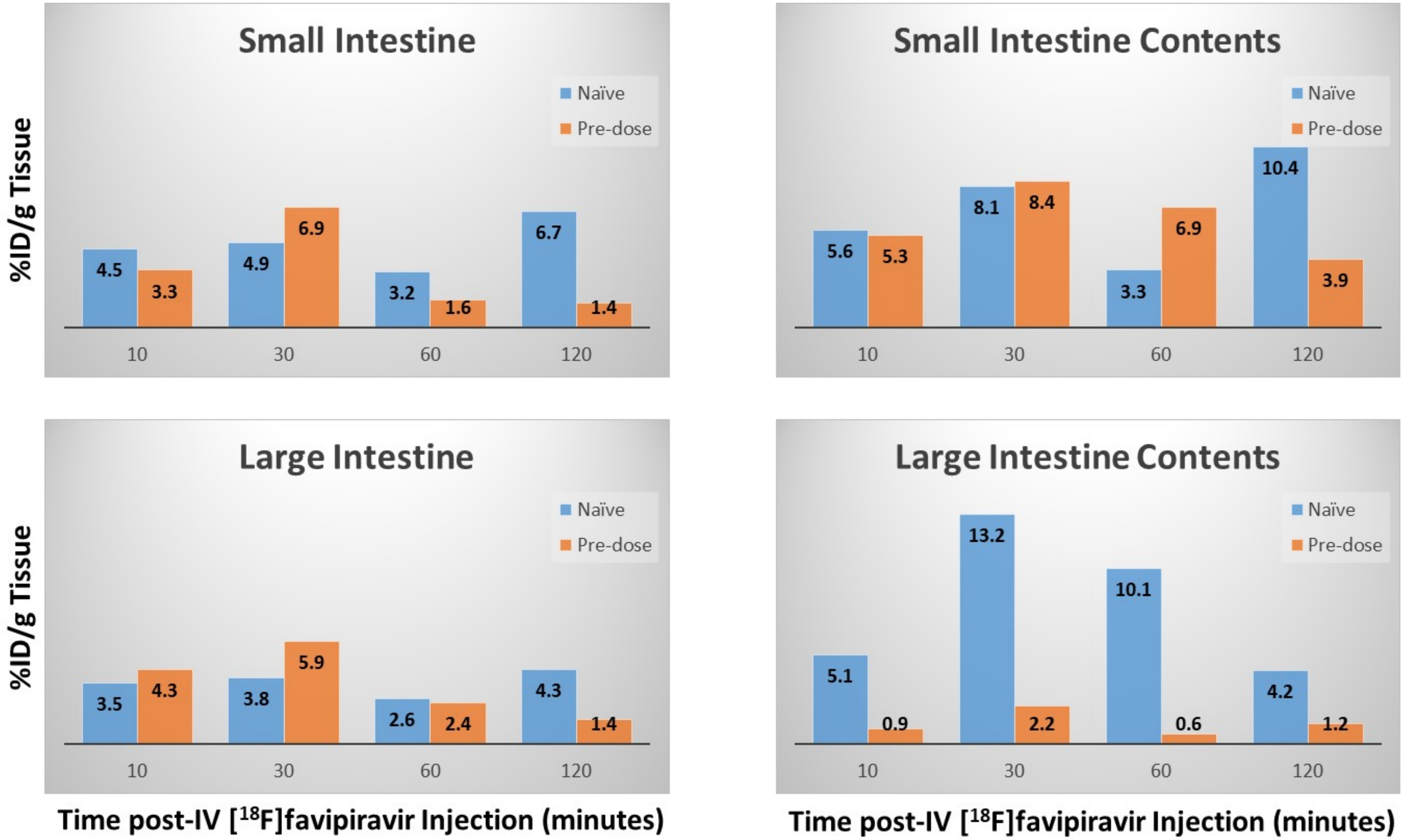

Supplementary Figure 3: Quantification of the temporal changes and [^{18}F]favipiravir levels in the small and larger intestine and their respective contents by gamma counting in naïve and pre-dosed mice. Value in each bar represents the %ID/g for the respective tissue.
